# Silver-Russell syndrome: clinical, neurodevelopmental and communication characteristics: clinical case studies

**DOI:** 10.1590/2317-1782/20212020273

**Published:** 2021-10-25

**Authors:** Eduarda Hanna Porto Ribeiro, Michele Dias Hayssi Haduo, Camila da Costa Ribeiro, Dionísia Aparecida Cusin Lamônica

**Affiliations:** 1 Departamento de Fonoaudiologia, Faculdade de Odontologia de Bauru, Universidade de São Paulo – FOB/USP - Bauru (SP), Brasil.

**Keywords:** Silver-Russell Syndrome, Neurodevelopmental Disorders, Phenotype, Child Language, Child Development, Speech Disorders, Cases Report

## Abstract

Silver Russell Syndrome (SRS) is a genetically heterogeneous condition with a clinical phenotype that includes intrauterine and postnatal growth restriction, craniofacial alterations, body asymmetries, low body mass index, and feeding difficulties. Alterations in motor development, global coordination, and speech are expected. The current study aims to present the syndrome, neurodevelopment, and communication characteristics of three male children diagnosed with the syndrome, aged 16, 18, and 44 months, respectively. Ethical principles were followed. An analysis of the medical records, aiming to collect information of the anamnesis, conducted with the guardians, and of the assessment carried out with the children was performed. The assessment was performed by applying the following instruments: Communicative Behavior Observation (CBO), Development Screening Test Denver-II (TSDD-II), and the Early Language Milestone Scale (ELMS). The survey of characteristics confirmed the SRS hypothesis; it was verified a delay in communicative behavior for all participants in CBO; in TSDD-II there was a delay in gross motor, fine motor-adaptive, language, and social personal skills. Scores below expectations were found for receptive auditory and expressive auditory functions, with receptive abilities more developed than expressive abilities, in ELM. The SRS deserves to be recognized by the scientific community, since the phenotypic characteristics and the data from the previous life allow the hypothesis of the syndrome to be raised, aiming at an early correct diagnosis and therapeutic planning that minimizes the harmful effects of this condition.

## INTRODUCTION

Silver-Russell Syndrome (SRS) is a genetically heterogeneous condition that affects 1 in every 30,000 to 100,000 children^(1)^. The classic phenotype includes intrauterine and postnatal growth retardation, hemihypertrophy, increased head circumference in relation to the body at birth, limb asymmetry, fifth finger clinodactyly, craniofacial disproportion, triangular face, feeding difficulties, and low body mass index (BMI )^(1-3)^.

Clinical diagnosis is performed through a classification consisted of at least four of the following characteristics: birth weight (≤-2 Standard Deviation); postnatal growth restriction, birth-related macrocephaly (head circumference ≥ 1.5 Standard Deviation above birth weight and/or length); facial characteristics, body asymmetry (leg length discrepancy with at least two asymmetrical body parts); feeding difficulties, low body mass index (BMI ≤ -2 Standard Deviation at 24 months)^(4,5)^. The clinical diagnosis is confirmed through molecular genetic tests^(1-5)^. However, in about 30% the molecular etiology is still unknown and, therefore, the clinical diagnosis is of fundamental importance ^(5)^ A study ^(4)^ showed that in about 60% of patients with a clinical diagnosis of SRS it is possible to identify a genetic cause and the most common disorders include loss of methylation on chromosome 11p15 (11p15 LOM; 30-60% of patients) and maternal uniparental disomy of the chromosome 7 (matUPD7; 5-10% of patients).

The vast majority of patients with SRS present feeding difficulties in early childhood, initiated by difficulty in sucking and, later, lack of appetite, food confusion, slow eating, and food aversion^(1)^. Gastrointestinal complications are common in children with SRS, including gastroesophageal reflux and esophagitis^(1,4)^. Dietary alterations are related to fragile oromotor control and may involve difficulties in coordinating the lips, tongue, and jaw^(4)^.

Generally, these individuals will have the intelligence in a normative pattern and the difficulties are concentrated in the global motor area and speech^(5-7)^. A study pointed out that the delay in motor development is apparent until the end of early childhood^(6)^. This delay may be related to low muscle mass, relative macrocephaly, and maturational alterations^(1,2,4)^.

Speech alterations are also common. Children with SRS, especially in the matUPD7 subgroup, present speech apraxia^(4)^ and learning difficulties, even if mild, as well as signs of Autism Spectrum Disorder^(4)^.

The aim of this clinical study is to present SRS, neurodevelopment, and communication characteristics of three male children with a clinical and genetic diagnosis of SRS.

## CLINICAL CASE PRESENTATION

Ethical principles were followed (CAE: 42356815.1.0000.5417). The legal guardians of the participants signed the Informed Consent Form (ICF). This is a cross-sectional study of medical records. From the information collected in the medical records, the following sequence of appointments was verified: initially, an anamnesis session with the guardian was held, followed by the application of assessment instruments: Communicative Behavior Observation (CBO)^(8)^, Denver Development Screening Test -II (TSDD-II)^(9)^, and Early Language Milestone Scale (ELM)(10). The age of the participants ranged from 16 to 44 months (P1 aged 16 months, P2 18 months, and P3 44 months).

In CBO^(8)^, communication skills are verified, which include the following categories: Communicative intention; Interaction with the examiner; Eye contact; Start of dialogue; Maintenance of dialogue; Vocalizations; Production of words; Production of phrases with more than two elements; Understanding of simple orders; Carrying out simple orders; Understanding of complex orders; Carrying out complex orders; Narrative; Symbolic play; Attention Time and the Functions of Informing, Protesting, Requesting, Offering and Imitating. A session of about thirty minutes was filmed for further analysis. In this study, descriptive analysis was used.

TSDD-II^(9)^ is a developmental screening scale for children aged zero to six years, which assesses performance in the following skills: Personal-Social (PS), Language (LG), Fine Motor-Adaptive (FMA), and Gross Motor (GM), with 125 items distributed for this purpose. Its application is carried out through direct testing of skills, observation of the behavior, and consideration of the history reported by parents. During instrument application, the child's age is initially calculated in months and then a vertical line is drawn in the recording protocol. Procedures relating to this age group are applied to all areas, following the instrument's application rules. After application, new performance lines are drawn for each of the areas evaluated, taking into account the last skill that the child successfully performed. Thus, four performance lines are obtained, corresponding to each of the skills assessed. The interpretation takes into account the age of the performance line obtained when compared to the chronological age line. In this way, performance can be classified as normal, at-risk, or delayed.

In ELM^(10)^, the Auditory Receptive (AR), Auditory Expressive (AE), and Visual (V) functions are verified. Forty-one behaviors are arranged on a single sheet, in graph form, so that you can locate each item and the month in which a particular skill should start. The scale presents a graph indicating the values of 25%, 50%, 75%, and 90% as representative of the percentages of children who would reach the skill tested during the scale validation process, within each age group. Initially, a vertical line is drawn across the scale, exactly at the child's chronological age. All items that cross the age line in all roles are checked. The three items of success and failure in each of the functions must be identified. If the 75% value in the ceiling item exceeds the child's chronological age, it is considered to have passed the ELM scale. The performance line can be drawn for each function assessed after analyzing the child's performance.

### Characterization of the cases

**P1**: The subject, male, 2nd child of a non-consanguineous couple, aged 16 months at the time of assessment. During the gestational follow-up, it was verified, through ultrasound exams, difficulties in the growth of the fetus. Birth by cesarean delivery, in the 37th gestational week, due to the change in intrauterine growth (sic). He was born measuring 45 cm, weighing 2270 grams, 32 cm of head circumference, and Apgar score of 10/10 in the first and fifth minutes, respectively. The child did not show normal growth and weight gain at birth and, nowadays, he still has scores below the standard deviation for his age group in height and weight measurements. He underwent speech therapy during the first six months of life, due to difficulties in feeding (sucking). During the assessment period, he was followed up with a nutritionist (sic). Presents as current feeding difficulties: lack of appetite, agitation at mealtimes, and slow eating. He presented cervical balance at around 3 months, sat unsupported at 8 months, crawled at 10 months, started to stand up at 12 months, and take the first steps at 1 year and four months. He started talking at 12 months being his first word “mommy”. The mother informs that the son understands everything that is said and that he is very smart, despite being quiet, and that his speech is not evolving, as he continues with only one word in his vocabulary “mommy”. He makes use of gestures (points and smiles when he wants something looking for what he wants). Her main complaint is the difficulty in feeding, as he has difficulty growing and gaining weight. He does not go to school. During the speech therapy evaluation, the evaluation by a geneticist was indicated. The clinical history indicated the main characteristics necessary for the clinical diagnosis of SRS. The genetic test result indicated SRS by 11p15 (loss of methylation of the ICR1 imprinting control region).
**P2:** The subject, male, 4th child of a non-consanguineous couple, 18 months old at the time of assessment. Pregnancy was uneventful, but there was a reduction in fetal growth. He was born by cesarean section, in the 38th gestational week, weighing 1860 grams, height of 45cm, head circumference of 35cm, and Apgar 8/9, in the first and fifth minutes, respectively. He showed cervical balance at 4 months; sat without support at 8 months; started walking at 15 months of age and at the time of the assessment it was still in process, that is, he was able to stand up and take a few steps; first words occurred at 18 months (mama). He had breastfeeding difficulties (breast milk offered in a cup for 2 months) and made use of food supplements. He has no interest in food (sic). He underwent a speech therapy assessment, aiming to improve his diet, and received guidance for three months. Follow up with a nutritionist. He was diagnosed with gastroesophageal reflux. Mom reports that he is smart, that he understands everything, but that he only speaks mommy and sounds of protest (crying and screaming). He uses gestures (no, he comes and points when he wants something). He does not go to school. Difficulty in growing up: he is small compared to his siblings (sic). He was indicated for evaluation with a geneticist when he started the speech-language diagnosis process. The clinical history points to the main characteristics necessary for the clinical diagnosis of SRS. The genetic test result indicated SRS by 11p15 LOM.
**P3:** The subject, male, 1st child of a non-consanguineous couple, 44 months old at the time of assessment. During pregnancy, due to decreased heart rate, reduced nutrient absorption, reduced growth, and decreased fetal activity, the mother was hospitalized from the 35th gestational week. He was born in the 39th gestational week of cesarean delivery, weighing 2165 grams, height 41 centimeters, head circumference 34 centimeters, and Apgar 8/10 in the first and fifth minutes, respectively. He spent 8 days in the incubator to gain weight, he had difficulties in sucking, both from the breast and from the baby bottle. During this period, he choked on the milk and spent 10 days in the Intensive Care Unit. At this time he received speech therapy orientation (sic). After this period he started to undergo treatment with a nutritionist. He showed cervical balance at 7 months; sat without support at 1 year and 2 months; first steps at 2 years and 6 months. The first meaningful words occurred at 24 months and at 44 months he still speaks few single words. The mother informed that because he is small, the people around him and at school tend to treat him like a baby. She believes that this behavior of people has an impact on her child's development, as he is overprotected (sic). The mother reported that the son has no appetite and that the meals are “arduous”, he has difficulty changing the menu and eats very slowly. He underwent physiotherapy treatment for eighteen months and with the speech therapist during his hospital stay. She informed that her son is still being followed up with an orthopedist, as he has a delay in bone age, which is around 1 year and 8 months. The panoramic radiograph of the entire spine showed: normal bone texture and vertebral bodies; right-hand convex lumbar scoliosis; lack of fusion of the L5-S1 posterior arch. He underwent an evaluation with a geneticist who indicated a molecular test, which has not been done yet. The clinical history suggested the main characteristics necessary for the clinical diagnosis of SRS, which was confirmed by a geneticist.

Chart 1 presents the main clinical characteristics of SRS and the characteristics verified in the studied cases.

**Chart 1:** Clinical Characteristics of SRS

Clinical Characteristics of SRS	P1	P2	P3
Intrauterine growth retardation	+	+	+
Increased head circumference at birth	+	+	+
Postnatal growth deficit	+	+	+
Late closing of fontanelles	-	+	NR
Prominent forehead	+	+	+
Triangular face	+	+	+
Asymmetry of members	+	+	+
Clinodactyly of the 5th finger	+	+	+
Syndactyly of the toes	-	-	+
Ripples (grooves) in the shoulders	+	+	-
Micrognathia	+	+	+
Low Body Mass Index (BMI)	+	+	+
Excessive sweating	+	-	-
Low implantation and/or posterior rotation of the ears	+	+	+
Mouth corners turned down	+	+	+
Irregular teeth	+	+	+
Male genitalia abnormalities	-	-	+
Prominent heels	+	+	+
Scoliosis and/or kyphosis	-	NR	+
Feeding difficulties	+	+	+
Difficulty growing and gaining weight	+	+	+
Motor Delay	+	+	+
Speech Delay	+	+	+

**Caption**: + = observed characteristic; - = characteristic not observed; NR = not reported

Table 1 shows the results obtained in CBO^(8)^.

**Table 1:** CBO Results

**PARTICIPANTS**	**CBO Result**
**P1**	Good interaction with evaluator; communicative intention, interest in toys; follow simple orders in immediate and concrete contexts. Made use of conventional non-symbolic gestures, points to what he wants, and smiles. Gave functionality only to the car. Presented rare non-articulated vocalizations and protest sounds, attention span in restricted self-interest situations.
**P2**	Good interaction with evaluator; communicative intention, interest in toys; follow simple orders in immediate and concrete contexts, always asking his mother. Look at people when they talk to him, has protest reactions (screaming, crying, gestures) when he wants something. Made use of conventional gestures (bye), spoke only mommy and produced sounds of protest (crying and screaming). Gave functionality to the ball and car. Showed attention span in restricted situations of self-interest and notions of presence and absence of objects.
**P3**	Good interaction with evaluator; communicative intention, interest in toys; eventually follow simple orders in immediate and concrete contexts. Respond unsystematically to request, comment, or when called upon. Comprehend situational orders with an action, accompanied by gestures (“send a kiss”). Explore objects quickly and superficially; has repetitive behaviors. Just said Mom. Presented vocalizations with intonation of the language (jargon). Presented conventional non-symbolic gestures (pointing, nodding, and a “come here” gesture). Protest. Reduced attention span.

The description of the observation of the communicative behavior of P1, P2, and P3 pointed to a lower performance than expected, considering the expectations of the chronological age groups of each of the participants.

Table 2 shows the performance of participants in TSDD-II^(9)^ in months.

**Table 2:** Performance of participants in TSDD-II in months

	**P1**	**P2**	**P3**
**Gross Motor**	12	14	30
**Fine Motor-Adaptive**	12	12	30
**Language**	14	12	24
**Personal-Social**	12	14	30

All participants had lower scores than expected for their age group in gross motor, fine motor-adaptive, language, and personal-social skills.

Table 3 shows the performance of the participants in ELM^(10)^ , regarding the data obtained in the performance line (in months).

**Table 3:** ELM results for the three participants

**Participantes**	**P1**	**P2**	**P3**
**Auditory Receptive Function**	16	18	28
**Auditory Expressive Function**	10	10	15
**Visual Function**	16	18	18*

* ELM in visual function assesses up to 18 months

The indices obtained in the auditory receptive function are adequate for the age group for P1 and P2. All participants had lower scores for their age group in auditory expressive function.

## DISCUSSION

SRS involves a wide variety of phenotypes and comorbidities^(1-7)^ The knowledge of the history and phenotypic clinical characteristics can favor the early diagnosis and interventional procedures of extreme relevance for the improvement of the clinical conditions and quality of life of these individuals and their families. Normally, the speech therapist acts early with these patients due to feeding difficulties started after birth (difficulty in sucking, breastfeeding and/or gastroesophageal reflux). This was what happened with the cases reported here, however, they received care only in the first months of life and were not followed up regarding development. It is noteworthy, as verified in the history of the participants, that the complaints about feeding are still present, which reinforces the need for speech therapy, in order to intervene in oral motor coordination and oral functions, in addition to nutritional issues.

Participants in this study were indicated for genetic evaluation during the speech-language diagnosis process at the ages of 16, 18, and 44 months, respectively. In this way, knowing the clinical phenotype of patients with SRS can favor, as a team member, the referral for genetic evaluation and better conduction of the therapeutic process, since feeding, weight gain and growth difficulties and alterations in oral functioning for feeding and speaking^(1,4,5)^, as well as the delay in motor development, are predicted in this syndrome.

From the neurodevelopmental point of view, the motor delay is predicted and is generally related to the reduction in muscle mass, relative macrocephaly, and neurophysiological brain immaturities^(1-5)^. This delay is best seen in early childhood^(6)^. The presence of motor disorders negatively influences the child's global development, reducing the possibility of extending motor experiences that, if integrated and significant, allow to explore the environment and to develop and organize the acquired knowledge^(11)^. Changes in global coordination are also foreseen^(2)^. The participants in this study had a delay in neuropsychomotor development (visualized by the history and predicted in the SRS characteristics, Chart 1) and scores in the gross motor and fine motor-adaptive areas lower than their chronological age (Table 2), confirming the clinical findings predicted in this syndrome^(2,5-7)^.

Although no information was found in the literature, specifically regarding language development, motor delay can interfere in the relationships the child establishes with people, events and environment, causing difficulties in language development. It is noteworthy that individuals with SRS generally present intellectual abilities in normative standards^(5-7)^. However, this does not mean that the processing of information may be taking place satisfactorily, as they may present changes in the attention span, interfering with the entire learning process. Attention enables the selection of information and is present in practically all actions and fundamental processes for child development^(12,13)^. Attentional changes can lead to disorganization of daily activities and are related to low performance in learning processes^(13-14)^, which can interfere with language development. Attention exerts its influence on the brain, modulating the activity of the neural systems involved in information processing, in order to facilitate the processing of information in the assisted channel, while processing in irrelevant channels is inhibited, which favors learning^(13-14)^.

A study pointed to mild learning difficulties^(4)^ and, therefore, longitudinal monitoring of communicative development is essential. Participants in this study present delay in language development (Table 1), and the receptive area is more developed than the expressive area (Table 3). This fact is presented in the literature since speech delay is reported as a high occurrence in this syndrome^(4-7)^. Speech motor control allows a flexible, fast, and accurate coordination of speech articulators to achieve a motor goal^(15)^. However, in SRS, alterations in oral motor control are already observed in feeding functions^(1,4,5)^. A study presented the possibility of occurrence of speech apraxia in childhood, depending on the etiology of SRS^(4)^. The participants in this study, due to maternal complaints and the results of the assessments (Tables 1, 2, and 3), are delayed in the development of language and, mainly, in expressive skills. The early detection of language and speech delays is based on knowledge of normative development patterns^(9-11)^ and professionals should be aware of the indication of early interventional procedures. All mothers reported that their children did not babble and another complaint, in addition to feeding issues and weight gain, is that despite understanding everyday contexts, speech does not evolve. In this sense, there is a need for clinical follow-up over time to analyze the evolution of these skills.

SRS is a genetically heterogeneous condition, well described in the literature^(1-7)^, regarding physical phenotype, clinical and genetic diagnosis. However, information on the evolution of oral motricity regarding feeding, speech and language functions is limited. Furthermore, longitudinal follow-up studies of individuals with SRS, regarding communicative skills, were not found in the literature. Studies of this nature can contribute to determine language phenotypes, describe communicative development trajectories in different syndromic conditions, and contribute to scientific development.

SRS is little known and deserves to be presented for recognition by the scientific community. The therapeutic follow-up of these children must be carried out by a team from different specialties and interventions must start as early as possible, to reduce the deleterious effects of the syndrome and to optimize the potential of these individuals.

## FINAL COMMENTS

Clinical history (intrauterine growth retardation, increased head circumference at birth, late closing of fontanelles, postnatal growth deficit, low body mass index, and excessive sweating), and phenotypic characteristics (triangular face, prominent forehead, micrognathia, asymmetry between limbs, clinodactyly, syndactyly, posterior rotation of the ears, mouth corners turned down, irregular teeth, scoliosis and/or kyphosis, prominent heels and male genitalia abnormalities), together with feeding difficulties indicate the clinical diagnostic hypothesis of SRS. The feeding difficulties started in the first breastfeeding make the speech therapist an extremely relevant professional in the care of these individuals. The participants presented neurodevelopmental alterations, mainly in the gross motor and fine motor-adaptive areas, and delayed communication skills, with better receptive than expressive language..
