# Supplementary material for: Silver-Russell syndrome: clinical, neurodevelopmental and communication characteristics: clinical case studies
Source: Codas. 2021 Oct 25;34(1):e20200273. doi: 10.1590/2317-1782/20212020273 (PMC9886125; doi:10.1590/2317-1782/20212020273)
Supplement: Translation

# Translation: Síndrome de Silver-Russell: Características clínicas, de neurodesenvolvimento e comunicação: Estudo de casos clínicos

**Authors:** Eduarda Hanna Porto Ribeiro, Michele Dias Hayssi Haduo, Camila da Costa Ribeiro, Dionísia Aparecida Cusin Lamônica

**Affiliations:** 1 Departamento de Fonoaudiologia, Faculdade de Odontologia de Bauru, Universidade de São Paulo – FOB/USP - Bauru (SP), Brasil.

**Keywords:** Síndrome de Silver-Russell, Transtornos do Neurodesenvolvimento, Fenótipo, Linguagem Infantil, Desenvolvimento Infantil, Distúrbios da Fala, Relatos de Casos

## Abstract

A Síndrome de Silver Russel (SSR) é uma condição geneticamente heterogênea com fenótipo clínico que inclui restrição do crescimento intrauterino e pós-natal, alterações craniofaciais, assimetrias corporais, baixo índice de massa corporal e dificuldades alimentares. Há expectativa de alterações do desenvolvimento motor, da coordenação global e de fala. O presente estudo tem como objetivo apresentar características da síndrome, do neurodesenvolvimento e comunicação de três crianças do sexo masculino, com diagnóstico da síndrome, na faixa etária de 16, 18 e 44 meses, respectivamente. Cumpriram-se os critérios éticos. Foi realizada análise de prontuário, com objetivo de coletar informações da anamnese realizada com os responsáveis, e da avaliação realizada com as crianças. A avaliação foi realizada por meio da aplicação dos seguintes instrumentos: Observação do Comportamento Comunicativo (OCC), Teste de Screening de Desenvolvimento Denver-II (TSDD-II) e o Early Language Milestone Scale (ELMS). O levantamento de características confirmou a hipótese da SSR; na OCC verificou-se atraso nos comportamentos comunicativos para todos os participantes; no TSDD-II verificou-se atraso nas habilidades motora grossa, motora fina-adaptativa, linguagem e pessoal social. Na ELM verificou-se escores aquém do esperado para as funções auditiva receptiva e auditiva expressiva com habilidades receptivas mais desenvolvidas do que as habilidades expressivas. A SSR merece ser reconhecida pela comunidade científica, uma vez que as características fenotípicas e os dados de vida pregressa, possibilitam que seja levantada a hipótese da síndrome, visando o diagnóstico correto precocemente e um planejamento terapêutico que minimize os efeitos deletérios desta condição.

## INTRODUÇÃO

A Síndrome de Silver-Russell (SSR) é uma condição geneticamente heterogênea que afeta 1 em cada 30.000 a 100.000 crianças^(1)^. O fenótipo clássico inclui retardo no crescimento intrauterino e pós-natal, hemihipertrofia, perímetro cefálico aumentado em relação ao corpo no nascimento, assimetria de membros, clinodactilia do quinto dedo, desproporção craniofacial, face triangular, dificuldades alimentares e baixo índice de massa corporal (IMC)^(1-3)^.

O diagnóstico clínico é realizado por meio de classificação composta por pelo menos quatro das seguintes características: peso ao nascer (≤-2 Desvio Padrão); restrição de crescimento pós-natal, macrocefalia relativa ao nascimento (perímetro cefálico ≥ 1,5 Desvio Padrão acima do peso e/ou comprimento ao nascer); características faciais, assimetria corporal (discrepância no comprimento das pernas com pelo menos duas partes corporais assimétricas); dificuldades alimentares, baixo índice de massa corporal (IMC ≤ -2 Desvio Padrão aos 24 meses)^(4,5)^. O diagnóstico clínico é confirmado por meio de testes genético moleculares^(1-5)^. Entretanto, em cerca de 30% a etiologia molecular ainda permanece desconhecida e, assim, o diagnóstico clínico é de fundamental importância ^(5)^. Um estudo ^(4)^ apresentou que em cerca de 60% dos pacientes com diagnóstico clínico de SSR é possível identificar uma causa genética e os distúrbios mais comuns incluem perda de metilação no cromossomo 11p15 (11p15 LOM; 30-60% dos pacientes) e dissomia materna uniparental do cromossomo 7 (matUPD7; 5-10% dos pacientes).

A grande maioria dos pacientes com SSR apresenta dificuldades alimentares na primeira infância, iniciadas pela dificuldade de sucção e, posteriormente a falta de apetite, confusão alimentar, alimentação lenta e aversão alimentar^(1)^. As complicações gastrointestinais são comuns em crianças com SSR, incluindo refluxo gastroesofágico e esofagite^(1,4)^. Alterações da alimentação estão relacionadas ao frágil controle oromotor e podem envolver dificuldades de coordenação dos lábios, língua e mandíbula^(4)^.

Geralmente, estes indivíduos terão a inteligência em padrão normativo e as dificuldades concentram-se na área motora global e fala^(5-7)^. Um estudo apontou que o atraso do desenvolvimento motor é aparente até o final da primeira infância^(6)^. Este atraso pode estar relacionado com a baixa massa muscular, a macrocefalia relativa e as alterações maturacionais^(1,2,4)^.

Alterações de fala também são comuns. Crianças com SSR, especialmente no subgrupo matUPD7, apresentam apraxia de fala^(4)^ e dificuldades de aprendizado, mesmo que leve, bem como sinais do Transtorno do Espectro Autista^(4)^.

Diante o exposto, o objetivo deste estudo clínico é apresentar características da SSR, do neurodesenvolvimento e comunicação de três crianças do sexo masculino, com diagnóstico clínico e genético da SSR.

## APRESENTAÇÃO DO CASO CLÍNICO

Cumpriram-se princípios éticos (CAE: 42356815.1.0000.5417). Os responsáveis legais dos participantes assinaram o Termo de Consentimento Livre e Esclarecido (TCLE). Trata-se de estudo de prontuário de tipologia transversal. Das informações coletadas no prontuário, verificou-se a seguinte sequência de atendimentos: inicialmente, foi realizada sessão de anamnese com o responsável, seguida da aplicação de instrumentos avaliativos: Observação do Comportamento Comunicativo (OCC)^(8)^, Teste de Screening de Desenvolvimento Denver-II (TSDD-II)^(9)^ e *Early Language Milestone Scale* (ELM)^(10)^. A idade dos participantes variou de 16 a 44 meses (P1 na faixa etária de 16 meses, P2 18 meses e P3 44 meses).

Na OCC^(8)^ são verificadas habilidades comunicativas, que englobam as seguintes categorias: Intenção comunicativa; Interação com avaliadora; Contato ocular; Inicio de diálogo; Manutenção do diálogo; Vocalizações; Produção de palavras; Produção de frases com mais de dois elementos; Compreensão de ordens simples; Realização de ordens simples; Compreensão de ordens complexas; Realização de ordens complexas; Narrativa; Brincar simbólico; Tempo de atenção e as Funções de informar, protestar, solicitar, oferecer e Imitar. Uma sessão de cerca de trinta minutos foi filmada para análise posterior. Foi utilizada, neste estudo, a análise descritiva.

O TSDD-II^(9)^ trata-se de uma escala de triagem do desenvolvimento para crianças de zero a seis anos, que avalia o desempenho nas seguintes habilidades: Pessoal-Social (PS), Linguagem (LG), Motor Fino-Adaptativo (MFA) e Motor Grosso (MG), com 125 itens distribuídos para esse fim. Sua aplicação é realizada por meio de testagem direta das habilidades, da observação do comportamento e da consideração do histórico relatado pelos pais. Na administração do instrumento, inicialmente, é calculada a idade da criança em meses e, em seguida, é traçada uma linha vertical no protocolo de registro. Aplicam-se procedimentos relativos a essa faixa etária para todas as áreas, seguindo as normas de aplicação do instrumento. Após a aplicação são traçadas novas linhas de desempenho para cada uma das áreas avaliadas, levando-se em consideração a última habilidade que a criança realizou com êxito. Dessa forma, obtém-se quatro linhas de desempenho, correspondentes a cada uma das habilidades avaliadas. A interpretação leva em conta a idade da linha de desempenho obtida quando comparada com a linha da idade cronológica. Desta forma, o desempenho pode ser classificado como: normal, risco ou atraso.

Na ELM^(10)^ verifica-se as funções Auditiva Receptiva (AR), Auditiva Expressiva (AE) e Visual (V). Quarenta e um comportamentos estão dispostos em folha única, na forma de gráfico, de maneira que se possa localizar cada item e o mês no qual determinada habilidade deve ter seu início. A Escala apresenta um gráfico indicando os valores de 25%, 50%, 75% e 90% como representativos de porcentagens de crianças que atingiriam a habilidade testada durante o processo de validação da escala, dentro de cada faixa etária. Inicialmente, traça-se uma linha vertical em toda a escala, exatamente na idade cronológica da criança. São verificados todos os itens que cruzam a linha da idade em todas as funções. Devem-se identificar os três itens de sucesso e de fracasso em cada uma das funções. Se o valor de 75% no item de teto exceder a idade cronológica da criança, considera-se que passou na escala ELM. Pode-se traçar a linha de desempenho em cada função avaliada após a análise do desempenho da criança.

### Caracterização dos casos

**P1:** O propósito, do gênero masculino, 2ª filho de casal não consanguíneo e com 16 meses na época da avaliação. Durante o acompanhamento gestacional, verificou-se por meio de exames de ultrassonografia dificuldades do crescimento do feto. Nascimento por parto cesariana, na 37ª semana gestacional, por conta da alteração do crescimento intrauterino (sic). Nasceu medindo 45 cm, com peso de 2270 gramas, 32 cm de perímetro cefálico e Apgar de 10/10 no primeiro e quinto minuto, respectivamente. A criança não apresentou crescimento e aumento de peso normal ao nascimento e, nos dias atuais, continua com escores abaixo do desvio padrão para sua faixa etária nas medidas de altura e peso. Fez acompanhamento fonoaudiológico nos primeiros seis meses de vida, devido a dificuldades para se alimentar (sucção). No período da avaliação realizava acompanhamento com nutricionista (sic). Apresenta como dificuldades alimentares atuais: falta de apetite, agitação nos horários de refeição e alimentação lenta. Apresentou equilíbrio cervical por volta de 3 meses, sentou sem apoio aos 8 meses, engatinhou aos 10 meses, começou a ficar em pé com 12 meses e a dar os primeiros passos com 1 ano e quatro meses. Começou a falar com 12 meses, sendo sua primeira palavra “mamãe”. Mãe informa que o filho compreende tudo o que é dito e que é bem esperto, apesar de ser quieto, e a fala não estar evoluindo, pois continua apenas com uma palavra em seu vocabulário “mamãe”. Faz uso de gestos (aponta e sorri quando quer algo olhando para o que deseja). Sua queixa principal é a dificuldade de alimentação, pois tem dificuldade para crescer e ganhar peso. Não frequenta escola. Durante a avaliação fonoaudiológica foi indicada a avaliação por geneticista. A história clínica indicou as principais características necessárias para o diagnóstico clínico da SSR. O resultado do exame genético indicou SSR por 11p15 (perda de metilação da região de controle de *imprinting* ICR1).
**P2:** O propósito, do gênero masculino, 4ª filho de casal não consanguíneo, de 18 meses na época da avaliação. Gravidez sem intercorrências, porém verificou-se redução do crescimento fetal. Nasceu de parto cesariana, na 38ª semana gestacional, com peso de 1860 gramas, estatura de 45cm, perímetro cefálico de 35cm e Apgar 8/9, no primeiro e quinto minuto, respectivamente. Apresentou equilíbrio cervical aos 4 meses; sentou sem apoio aos 8 meses; iniciou marcha aos 15 meses e no momento da avaliação ainda estava em processo, ou seja, conseguia ficar em pé e dava poucos passos; primeiras palavras ocorreu aos 18 meses (mama). Apresentou dificuldades para mamar (leite materno oferecido em copinho por 2 meses) e fez uso de complemento alimentar. Não tem interesse por comida (sic). Fez avaliação fonoaudiológica, visando melhorar alimentação, e recebeu orientações por três meses. Faz acompanhamento com nutricionista. Teve diagnóstico de refluxo gastroesofágico. Mãe relata que é esperto, que compreende tudo, mas fala apenas mamãe e sons de protesto (choro e grito). Usa gestos (não, vem e aponta quando quer algo). Não frequenta escola. Dificuldade de crescimento: é pequeno em relação aos irmãos (sic). Foi indicado para avaliação com geneticista, quando iniciou processo de diagnóstico fonoaudiológico. A história clínica sinaliza para principais características necessárias para o diagnóstico clínico da SSR. O resultado do exame genético indicou SSR por 11p15 LOM.
**P3:** O propósito, do gênero masculino, 1^o^ filho de casal não consanguíneo, de 44 meses na época da avaliação. Durante a gestação, devido a diminuição de batimentos cardíacos, redução da absorção dos nutrientes, redução de crescimento e diminuição da atividade fetal, a mãe ficou internada desde a 35ª semana gestacional. Nasceu na 39ª semana gestacional de parto cesáriana, com peso de 2165 gramas, estatura de 41 centímetros, perímetro cefálico de 34 centímetros e Apgar 8/10 no primeiro e quinto minuto, respectivamente. Ficou 8 dias na incubadora para ganhar peso, apresentava dificuldades para sugar, tanto do peito quanto na mamadeira. Neste período, se engasgou com o leite e passou 10 dias na Unidade de Terapia Intensiva. Nesta época recebeu orientações fonoaudiológicas (sic). Após este período passou a fazer tratamento com nutricionista. Apresentou equilíbrio cervical aos 7 meses; sentou sem apoio com 1 ano e 2 meses; primeiros passos com 2 anos e 6 meses. As primeiras palavras com significado ocorreram com 24 meses e, aos 44 meses, ainda fala poucas palavras isoladas. A mãe informou que pelo fato de ser pequeno as pessoas do convívio e da escola tendem a tratá-lo como bebê. Acredita que este comportamento das pessoas traz impacto para o desenvolvimento de seu filho, pois é superprotegido (sic). A mãe relatou que o filho não tem apetite e que as refeições são “custosas”, tem dificuldade com mudança de cardápio e come muito devagar. Fez tratamento com fisioterapia por dezoito meses e com fonoaudiólogo durante a estadia no hospital. Informou que o filho ainda realiza acompanhamento com ortopedista, pois apresenta atraso na idade óssea, que está em torno de 1 ano e 8 meses. A radiografia panorâmica de coluna total apontou: textura óssea e corpos vertebrais normais; escoliose lombar destro convexa; falta de fusão do arco posterior de L5-S1. Realizou avaliação com geneticista que indicou exame molecular, ainda não realizado. A história clínica sugeria as principais características necessárias para o diagnóstico clínico da SSR, que foi confirmado por geneticista.

O Quadro 1 apresenta as principais características clínicas da SRS e as características verificadas nos casos estudados

**Quadro 1:** Características Clínicas da SSR

Características Clínicas da SSR	P1	P2	P3
Retardo do crescimento intrauterino	+	+	+
Perímetro cefálico aumentado ao nascimento	+	+	+
Déficit de crescimento pós-natal	+	+	+
Fechamento tardio das fontanelas	-	+	NR
Fronte proeminente	+	+	+
Face triangular	+	+	+
Assimetria dos membros	+	+	+
Clinodactilia do 5o dedo	+	+	+
Sindactilia dos dedos dos pés	-	-	+
Ondulações (sulcos) nos ombros	+	+	-
Micrognatia	+	+	+
Baixo Índice de Massa Corporal (IMC)	+	+	+
Sudorese excessiva	+	-	-
Baixa implantação e/ou rotação posterior das orelhas	+	+	+
Cantos da boca voltados para baixo	+	+	+
Dentes irregulares	+	+	+
Anormalidades de genitália masculina	-	-	+
Calcanhares proeminentes	+	+	+
Escoliose e/ou cifose	-	NR	+
Dificuldades alimentares	+	+	+
Dificuldade para crescer e ganhar peso	+	+	+
Atraso motor	+	+	+
Atraso na fala	+	+	+

**Legenda**: + = característica observada; - = característica não observada; NR = não relatado

A Tabela 1 apresenta os resultados obtidos na OCC^(8)^

**Tabela 1:** Resultados da OCC

**PARTICIPANTES**	**RESULTADO OCC**
**P1**	Boa interação com avaliador; intenção comunicativa, interesse por brinquedos; acata ordens simples em contextos imediatos e concretos. Fez uso de gestos não simbólicos convencionais, aponta para o que deseja e sorri. Deu funcionalidade somente para carro. Apresentou raras vocalizações não articuladas e sons de protesto, tempo de atenção em situações restritas de interesse próprio.
**P2**	Boa interação com avaliador; intenção comunicativa, interesse por brinquedos; acata ordens simples em contextos imediatos e concretos, sempre solicitando a mãe. Olha para as pessoas quando falam com ele, apresenta reações de protesto (gritos, choro, gestos) quando quer algo. Fez uso de gestos convencionais (tchau), falou apenas mamãe e produziu sons de protesto (choro e gritos). Deu funcionalidade à bola e carro. Apresentou tempo de atenção em situações restritas de interesse próprio e noções de presença e ausência de objetos.
**P3**	Boa interação com avaliador; intenção comunicativa, interesse por brinquedos; eventualmente acata ordens simples em contextos imediatos e concretos. Responde de forma assistemática a solicitação, comentário ou quando chamado. Compreende ordens situacionais com uma ação, acompanhadas por gestos (“mande um beijo”). Explora os objetos de modo rápido e superficial; tem comportamentos repetitivos. Só falou mamãe. Apresentou vocalizações com entonação da língua (jargão). Apresentou gestos não simbólicos convencionais (apontar, negar com a cabeça e gesto de “vem cá”). Protesta. Tempo de atenção reduzido.

A descrição da observação do comportamento comunicativo de P1, P2 e P3 apontou para desempenho inferior ao esperado, considerando as expectativas das faixas etárias cronológicas de cada um dos participantes.

A Tabela 2 apresenta o desempenho dos participantes no TSDD-II^(9)^ em meses.

**Tabela 2:** Desempenho dos participantes no TSDD-II em meses

	**P1**	**P2**	**P3**
**Motor grosso**	12	14	30
**Motor fino-adaptativo**	12	12	30
**Linguagem**	14	12	24
**Pessoal-Social**	12	14	30

Todos os participantes obtiveram escores abaixo do esperado para sua faixa etária nas habilidades motora grossa, motora fina-adaptativa, linguagem e pessoal-social.

A Tabela 3 apresenta o desempenho dos participantes na ELM^(10)^, quanto aos dados obtidos na linha de desempenho (em meses).

**Tabela 3:** Resultado da ELM para os três participantes

**Participantes**	**P1**	**P2**	**P3**
**Função Auditiva receptiva**	16	18	28
**Função Auditiva expressiva**	10	10	15
**Função Visual**	16	18	18*

* A ELM na função visual avalia até 18 meses

Os índices obtidos na função auditiva receptiva estão adequados para a faixa etária para P1 e P2. Todos os participantes obtiveram escores inferiores para a faixa etária na função auditiva expressiva.

## DISCUSSÃO

A SSR envolve uma ampla variedade de fenótipos e comorbidades^(1-7)^. O conhecimento do histórico e características clínicas fenotípicas pode favorecer o diagnóstico precoce e procedimentos interventivos de extrema relevância para a melhoria das condições clínicas e qualidade de vida destes indivíduos e suas famílias. Via de regra, o fonoaudiólogo atua precocemente com estes pacientes pelas dificuldades de alimentação iniciadas após o nascimento (dificuldade de sucção, amamentação e/ou refluxo gastroesofágico). Isto foi o que ocorreu com os casos aqui relatados, entretanto, receberam atendimentos somente nos primeiros meses de vida e não foram acompanhados quanto ao desenvolvimento. Cabe ressaltar, conforme verificado no histórico dos participantes, que as queixas quanto à alimentação ainda estão presentes, o que reforça a necessidade do acompanhamento fonoaudiológico, no intuito de intervir na coordenação motora oral e funções orais, para além das questões nutricionais.

Os participantes deste estudo foram indicados para avaliação genética durante a realização do processo de diagnóstico fonoaudiológico com as idades de 16, 18 e 44 meses, respectivamente. Desta forma, conhecer o fenótipo clínico de pacientes com a SSR pode favorecer, como membro de equipe, o encaminhamento para avaliação genética e melhor condução do processo terapêutico, uma vez que é previsto nesta síndrome as dificuldades alimentares, de ganho de peso e crescimento e as alterações do funcionamento oral para a alimentação e fala^(1,4,5)^, bem como o atraso do desenvolvimento motor.

Do ponto de vista do neurodesenvolvimento o atraso motor é previsto e geralmente está relacionado com a redução da massa muscular, macrocefalia relativa e imaturidades neurofisiológicas cerebrais^(1-5)^. Este atraso é melhor visualizado na primeira infância^(6)^. A presença de transtornos motores influencia negativamente no desenvolvimento global da criança, reduzindo a possibilidade de prolongar experiências motoras que, se integradas e significativas, possibilita explorar o meio ambiente e elaborar e organizar o conhecimento adquirido^(11)^. Alterações da coordenação global também são previstas^(2)^. Os participantes deste estudo tiveram atraso no desenvolvimento neuropsicomotor (visualizado pelo histórico e previstos nas características da SSR, Quadro 1) e escores nas áreas motora grossa e motora fina-adaptativa inferiores à sua idade cronológica (Tabela 2), confirmando os achados clínicos previstos nesta síndrome^(2,5-7)^.

Apesar de não ter sido encontradas informações na literatura, especificamente quanto ao desenvolvimento da linguagem, o atraso motor pode interferir nas relações que a criança estabelece com pessoas, eventos e ambiente, acarretando dificuldades para o desenvolvimento da linguagem. Cabe ressaltar que geralmente os indivíduos com SSR apresentam as habilidades intelectuais em padrões normativos^(5-7)^. Entretanto, isto não significa que o processamento das informações possa estar ocorrendo de forma satisfatória, pois podem apresentar alterações no tempo de atenção, com interferência em todo o processo de aprendizagem. A atenção possibilita a seleção da informação, e está presente em praticamente todas as ações e processos fundamentais para o desenvolvimento infantil^(12,13)^. Alterações atencionais podem levar à desorganização das atividades cotidianas e está relacionada ao baixo rendimento nos processos de aprendizagens^(13-14)^, dentre os quais pode trazer interferências para o desenvolvimento da linguagem. A atenção exerce sua influência no cérebro, modulando a atividade dos sistemas neurais envolvidos no processamento de informações, de modo a facilitar o processamento de informações no canal assistido, enquanto o processamento em canais irrelevantes é inibido, o que favorece a aprendizagem^(13-14)^.

Um estudo apontou para dificuldades leves de aprendizado^(4)^ e, desta forma, o acompanhamento longitudinal do desenvolvimento comunicativo é fundamental. Os participantes deste estudo apresentam atraso no desenvolvimento da linguagem, (Tabela 1), e a área receptiva está mais desenvolvida do que a expressiva (Tabela 3). Fato este apresentado na literatura, uma vez que o atraso de fala é relatado como alta ocorrência nesta síndrome^(4-7)^. O controle motor da fala permite uma coordenação flexível, rápida e precisa dos articuladores da fala para atingir uma meta motora^(15)^. Entretanto, na SSR as alterações do controle motor oral já são verificadas nas funções alimentares^(1,4,5)^. Um estudo apresentou a possibilidades de ocorrência de apraxia de fala na infância, dependendo da etiologia da SSR^(4)^. Os participantes deste estudo, pelas queixas maternas e resultados das avaliações (Tabelas 1, 2, e 3), estão com atraso no desenvolvimento da linguagem e, principalmente, das habilidades expressivas. A detecção precoce de atrasos na linguagem e na fala é baseada no conhecimento dos padrões normativos de desenvolvimento^(9-11)^ e os profissionais devem estar atentos para a indicação de procedimentos interventivos precoces. Todas as mães relataram que os filhos pouco balbuciaram e outra queixa, além das questões alimentares e ganho de peso, é que apesar de compreenderem contextos cotidianos a fala não evolui. Neste sentido, há necessidade de acompanhamento clínico ao longo do tempo para análise da evolução destas habilidades.

A SSR é uma condição geneticamente heterogênea, bem descrita na literatura^(1-7)^, quanto ao fenótipo físico, diagnóstico clínico e genético. Entretanto, as informações sobre evolução da motricidade oral quanto as funções de alimentação, fala e linguagem são limitadas. Além disso, estudos de acompanhamento longitudinal de indivíduos com SSR, quanto as habilidades comunicativas, não foram encontrados na literatura. Estudos desta natureza podem contribuir para determinar fenótipos de linguagem, descrever trajetórias de desenvolvimento comunicativo nos diferentes quadros sindrômicos e contribuir para o desenvolvimento científico.

A SSR é pouco conhecida e merece ser apresentada para o reconhecimento da comunidade científica. O acompanhamento terapêutico destas crianças deve ser realizado por equipe de diferentes especialidades e as intervenções devem iniciar o mais precocemente possível, com o intuito de reduzir os efeitos deletérios da síndrome e otimizar o potencial destes indivíduos.

## COMENTÁRIOS FINAIS

O histórico clínico (retardo do crescimento intrauterino, perímetro cefálico aumentado ao nascimento, fechamento tardio das fontanelas, déficit de crescimento pós-natal, baixo índice de massa corporal e sudorese excessiva), e as características fenotípicas (face triangular, fronte proeminente, micrognatia, assimetria entre os membros, clinodactilia, sindactilia, rotação posterior das orelhas, cantos da boca voltados para baixo, dentes irregulares, escoliose e/ou cifose, calcanhares proeminentes e anormalidades de genitália masculina), somadas as dificuldades alimentares indicam a hipótese diagnóstica clínico da SSR. As dificuldades alimentares iniciadas nas primeiras mamadas colocam o fonoaudiólogo como um profissional de extrema relevância no atendimento destes indivíduos. Os participantes apresentaram alterações do neurodesenvolvimento, principalmente nas áreas motora grossa e fina-adaptativa, e atraso nas habilidades comunicativas, com linguagem receptiva melhor que a expressiva.
